# Langerhans cell histiocytosis in adults: a case report and review of the literature

**DOI:** 10.18632/oncotarget.7892

**Published:** 2016-03-03

**Authors:** Cuihong Lian, Yuan Lu, Siyuan Shen

**Affiliations:** ^1^ Shenzhen Second People's Hospital, The First Affiliated Hospital of Shenzhen University, Shenzhen, Guangdong, China; ^2^ Nanshan People's Hospital of Shenzhen, Shenzhen, Guangdong, China

**Keywords:** histiocytosis, Langerhans cell, adults, clinical pathology

## Abstract

**Background:**

Langerhans cell histiocytosis (LCH) is a proliferative disease of histiocyte-like cells that generally affects children. Immunohistochemistry is essential to obtain the correct diagnosis, and treatment protocols are controversial.

**Objective:**

Langerhans cell histiocytosis (LCH) is easy to be misdiagnosed because of its various clinic features and laboratory results. This research focused on the clinicopathological, histopathological, immunohistochemical and other features of LCH and aimed to analyze LCH clinical features for improving diagnosis and decreasing misdiagnosis rate.

**Case report:**

A case of rare adult LCH was reported and the clinicopathological features were summarized by literature review. The multifocal form of this case includes diabetes insipidus, exophthalmos and mucocutaneous lesions in axillae and anogenital regions, such as infiltrated nodules, extensive coalescing, scaling, crusted papules and ulcerated plaques. The Langerhans cells diffusely infiltrated in the dermis and the tumor cells were positive for CD1a and S-100 expression. The diagnosis was Langerhans cell histiocytosis based on the pathological and immunohistochemical changes.

**Conclusion:**

LCH has high rate of misdiagnosis and definitive diagnosis depends on pathological biopsy and X-ray examination. The prognosis is related to the onset age and the quantity of affected organs. Although specific therapeutic approach hasn't been well established, combined chemotherapy for multisystem lesions and surgical operation or radiotherapy for unifocal lesions may improve the therapy.

## INTRODUCTION

Langerhans cell histiocytosis (LCH), characterized by intense and abnormal proliferation of bone marrow-derived histiocytes (Langerhans cells), is a rare disease of unknown pathogenesis, leading to its high rate of misdiagnosis and missed diagnosis [1]. Here we introduce a case of LCH by reviewing related literatures and focus on the clinical manifestations, histopathologic characteristics and differential diagnosis of similar dermatoses.

### Case report

**Figure 1:**
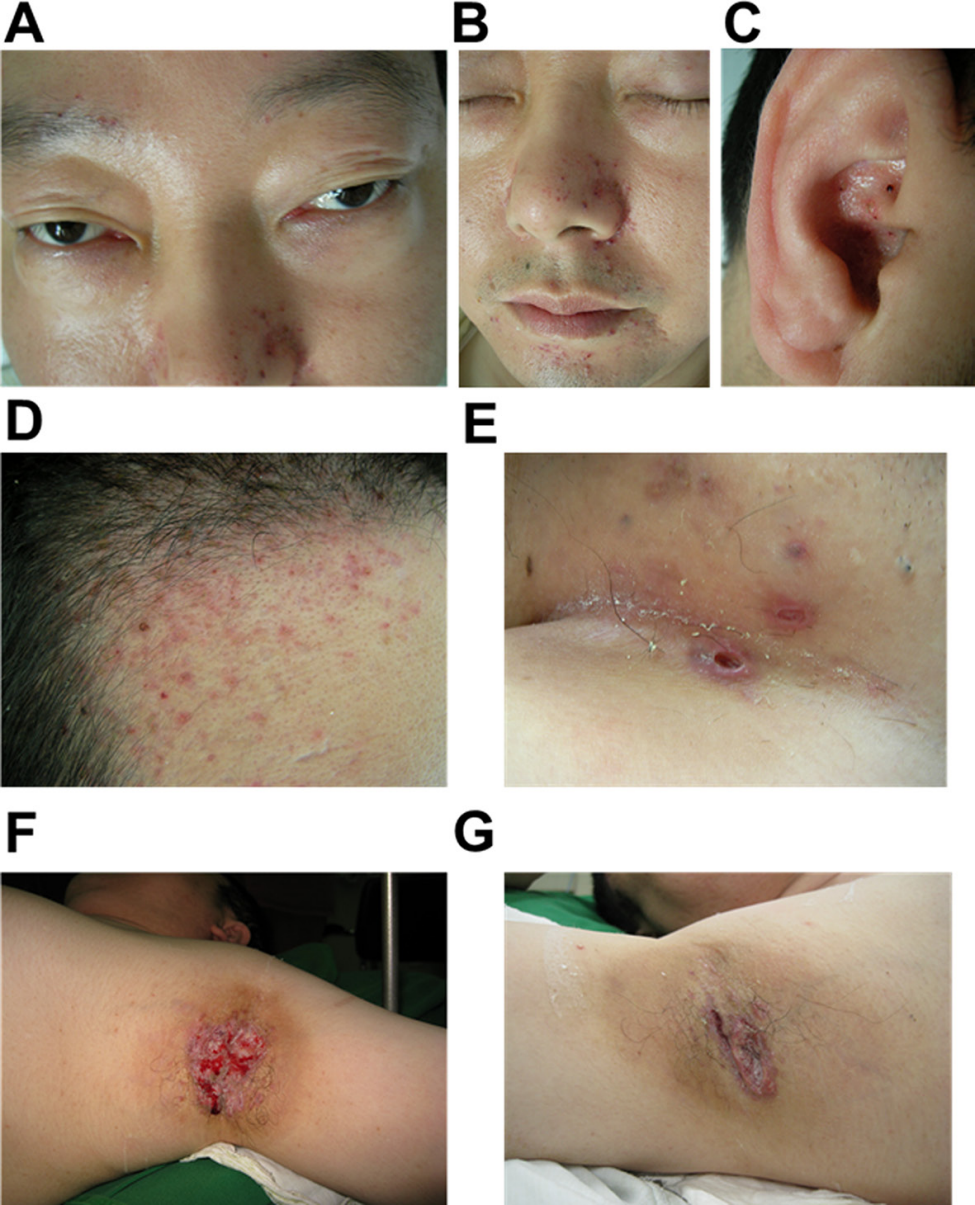
The clinical features of this patient (**A**) Exophthalmos. (**B**) Damage like seborrheic dermatitis on the face. (**C**) Damage like seborrheic dermatitis in the auricle. (**D**) Damage like seborrheic dermatitis over his scalp and face. (**E**) Nest-like ulcers in Bilateral groin, about soybean volume. The surface was dry, without secretion. There were also some sporadic pimples with scab. (**F**) Ulcer in the left oxter. (**G**) Ulcer in the right oxter.

A 34-year-old man complains of bilateral axillary ulcers with pain for half a year, and has chest congestion, weakness and polyuria for 3 years. In the past 3 years, the patient developed many weaknesses, including alopecia, sexual dysfunction, weight increment, chest congestion, mastauxy accompanied with pain, polydipsia and polyuria. One year ago, the patient got bilateral axillary ulcers which grew gradually. Besides, his head and face also appeared “red rash” together with pruritus. He was diagnosed as “multiple Boil”, “skin and soft tissue infections”, “skin ulcers investigation”, “hidradenitis”, “eczema”, “candidiasis”, “Hailey-Hailey disease (suspicious)”, “seborrheic dermatitis” and etc. by various hospitals. He was treated with anti-infection agents, debridement and topical drugs, including povidone-iodine, fusidic acid, mupirocin and oral medicines (cefaclor and azithromycin). After these treatments, symptoms had no significant improvement so the patient came to Department of Dermotology, Shenzhen Second People's Hospital, (Shenzhen, China) for further examination on April 13, 2015. The usage of the patient's information was approved by him and the hospital ethics committee. He was considered asymptomatic because of not having fever, weight lost or night sweats. His family history was unremarkable. Physical examination revealed that the patient was mentally normal, with an anemia outlook, a fatty figure, a moon face and exophthalmos (Figure 1A). Double lung breath sounds rough, and there were moderate rough moist rales. He had a bulging abdomen, and the liver was palpable 3 cm away from the right costal arch, with a medium texture and a sharp edge. Dermatologist examination revealed that light red or yellowish-white millet pimples were appeared on his scalp and face (area around the nose and forehead mainly). There were greasy yellow scales on the surface, and it showed hemorrhagic spots after been removed forcibly. In bilateral inguinal, there were nest-like ulcers, each of which is as large as soybean. The surface was dry and has some blood scabs on it but without secretion. We could see irregular ulcers with clear boundary in both axillary, about 5cm-long. The skin around the ulcers was red, swelling and tender, with white thin moss-like substance and granulation tissue. There was no obvious fester and specific smell (Figure 1B–1G).

**Figure 2:**
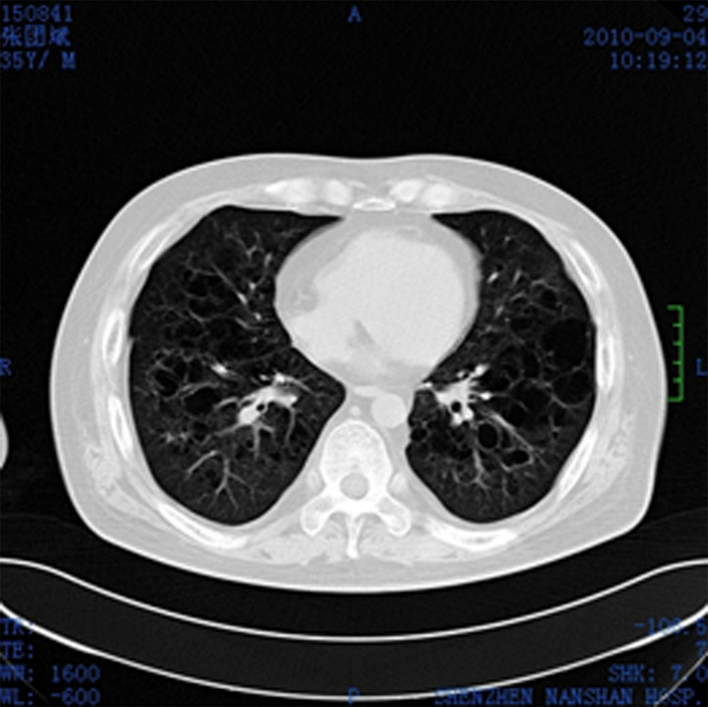
Brest CT plain scan Higher penetrance of lung fields, extensive unequal-sized flake or cystic bright shade, and incrassate shade of interlobular septum.

**Figure 3:**
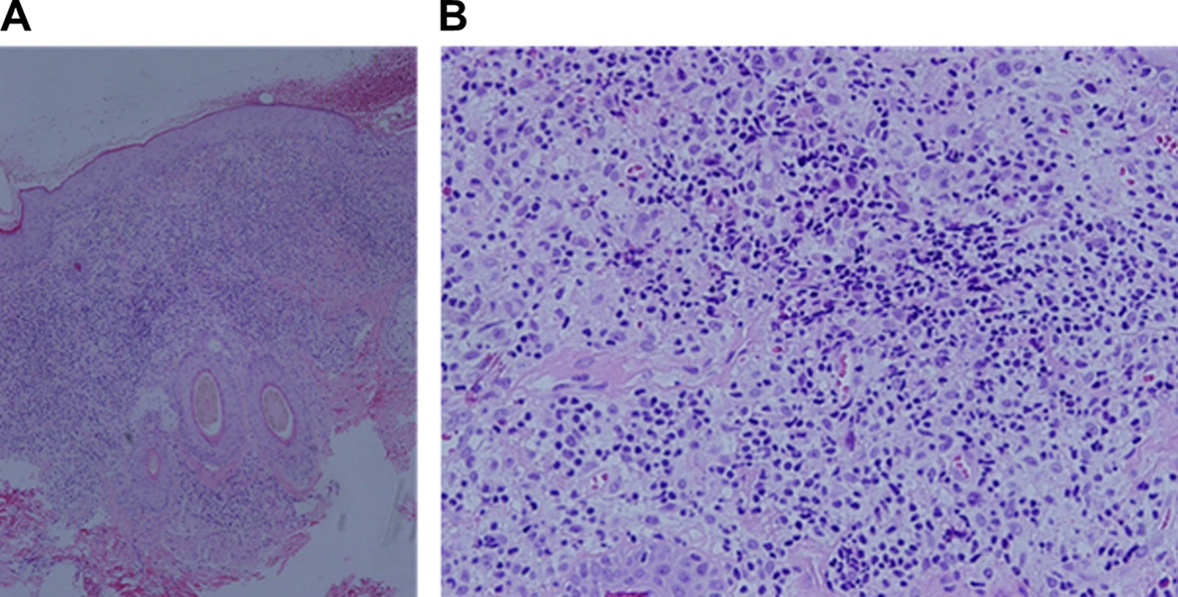
Lesion histopathological examination (**A**) Dermal papilla edema, with abundant hyperplastic large round cells, in which we could see nuclear groove, together with several eosinophile granulocytes and lymphocytes infiltrated. (HE × 100). (**B**) Abundant hyperplastic large round cells, in which we could see nuclear groove, together with several infiltrated eosinophile granulocytes and lymphocytes. (HE × 200).

**Figure 4:**
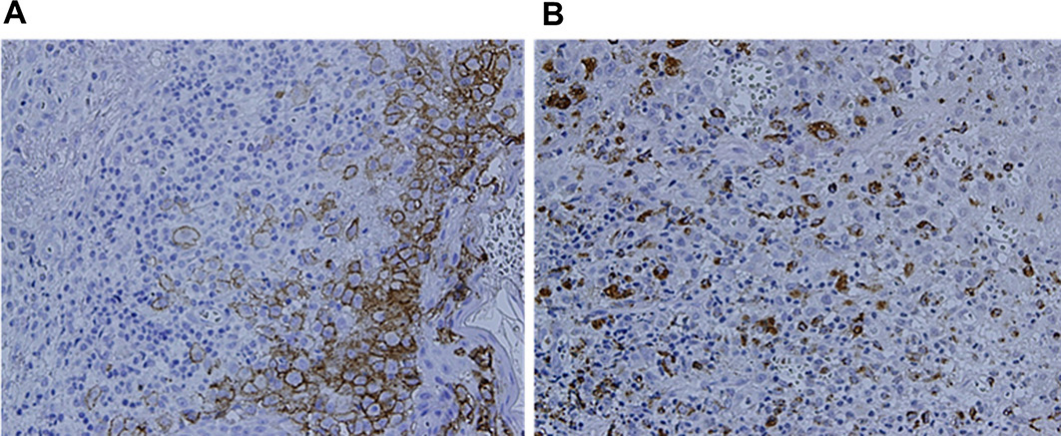
Immunohistochemical examination (**A**) CD1a (+) (SP × 400) (**B**) CD68 (+) (SP × 400).

Laboratory examination revealed that the patient's erythrocyte sedimentation rate (ESR) was 23 mm/1 h (≤ 15 mm/1 h) and CRP was 50 mg/L. Bone Marrow Examination revealed that the bone marrow nucleated cells containing 47% granulocytes composed of vacuoles and toxic particles and 41% erythrocytes composed of polychromatic erythroblast had obviously proliferated with an expanding pale area. TSH was 0.021 Miu/L (0.35–4.94), testosterone was 0.67 ng/ML, PRL was 57.60 ng/ML and cortisol was 12.70 ug/dl. Liver function showed that ALT was 194 U/L (≤ 40 U/L), AST was 135U/L (≤ 50 U/L) and LDH was 409 U/L (100–300 U/L). Lymphocyte subpopulation were composed of 26.30% NK cells, 9.57% CIK cells (a little higher), and 1.89% B cells (a little lower), which indicated a low-level humoral immunity. Pituitary MRI scan revealed that most of pituitary fossa showed a cerebrospinal fluid signal. The pituitary volume was significantly narrowed and was located at the sellar floor with a line sample. The infundibulum signal was thickened unequally with a change of empty sella. ECT bone scan showed irregular radioactive concentration shadow of the sternum, bilateral ilium and hip joint, which indicated an active metabolism of bone mineral. Bonchoalveolar lavage luid routine showed that total nucleated cells were 280 × 10^6^/L in which monocyte occupied 85% and coenocyte occupied 15%. The lavage fluid bacterial culture indicated the infection of viridans streptococcus. CD4/CD8 was declined (0.95%). The result of CD1α macrophage reaction was positive (+). CT plain scan showed a T2 high signal nodular shadow with a length of 8mm in the right eye orbital wall. Brest CT showed that there were higher penetrance of lung fields, extensive unequal-sized flake or cystic bright shade, and incrassate shade of interlobular septum (Figure 2). Abdomen B ultrasonography revealed that his liver was enlarged. The scalp lesion pathology showed dermal papilla edema and abundant hyperplastic large round cells, in which we could see nuclear groove, several eosinophile granulocytes and infiltrated lymphocytes (Figure 3A and 3B). Immunohistochemical examination (Figure 4A and 4B) showed that histioid cell cytoplasm and nucleus were CD1a (+) and histioid cell cytoplasm were CD68 (+). Pathology of the alar skin ulcers showed that there were diffuse patchy medium sized hyperplastic cells. The actively proliferating cells have folding and sagging nucleus, smooth and exquisite chromatin and thin nuclear membrane. The common expression pattern of these cells was S-100 (+), CD1a (+), CD68 (+), CD3 (−) and CD79a (−). About 20% cells express the cell proliferation marker Ki-67.

Based on the above results of breast CT, BAL, histopathological and immunohistochemical examinations, the patient had diabetes insipidus, bone damage and nodular shadows of hypothalamus. Take all these results into consideration, a diagnosis of Langerhans cell histiocytosis (Hand-Schuller-Christian Disease) was validated. Then we take the therapy of CHOPE (cytoxan, adriamycin, vincristine, prednisone and etoposide) together with oral isotretinoin, and we carried out the operation of “debridement, free skin transplantation and thigh skin graft operation” for him. The operation was successful and the wound healed well. After half a year's follow-up, his systemic symptom was reduced and rash was apparently disappeared. There was no recidivation of his ulcers in the axillary.

## DISCUSSION

Langenhans cell histiocytosis(LCH), also known as histiocytosis X(HX), is a group of hyperplastic cellular diseases of unknown causes. The Histolocyte Society renamed it as Langerhans cell histiocytosis [2] in 1987. Now LCH is divided into 3 types by a recognized classification method, including acute disseminated LCH, chronic multifocal LCH and chronic focal LCH. But more people divide it into 2 types according to the lesion scope: (1) Localized LCH: ① simplex rash, without affecting organs. ② simplex bone damage, with or without diabetes insipidus(DI), adjacent lymph nodes involvement or rash. ③ multiple bone damages, including more than one bones or one bone with more than two damages, with or without diabetes insipidus(DI), adjacent lymph nodes involvement or rash. (2) Disseminated LCH: ① the internal organs were involved, with or without diabetes insipidus(DI), adjacent lymph nodes involvement or rash, without dysfunction of lung, liver or hemopoietic system. ② apart from the above symptoms, the internal organs are involved as well, with dysfunction of lung, liver or hemopoietic system [3]. This classification is comprehensive and practical, and covers all kinds of LCH, thus providing a better promotion of the treatment and prognosis. So it is widely recommended by many scholars.

LCH can occur at any age, but mainly in children of 1∼4 year-old. The incidence of LCH in adults is 1–2 cases per million. Most LCH patients are males. The sex ratio (m:f) was 2:1. And its etiology remains unknown until now. With the development of modern molecular biology and genetics, as well as the clinical research in-depth, the disease is considered to be a kind of reactive hyperplastic disease. The occurrence and development of this disease is closely related to chromosomal instability and gene mutation. Tissue cells have the character of clonal proliferation, which indicates that the native lesion of the disease is tumor [4]. There are also some scholars who consider that LCH is associated with cytokine mediation, immunologic derangement, virus infection [5] and etc.

The diagnosis of LCH depends on clinical manifestation, iconography examination, lesion histopathology and electron microscopy. Pathological examination includes print and pathological biopsy and immunohistochemical examination. Pathological materials can be taken from the rash, lymph nodes, the tumor or the local lesions. Langerhans cells were accumulated in the epidermis by Light microscopy. The nucleus was folded within a groove, making it looks like coffee beans. Eosinophils and other inflammatory cells was sporadic round in shape. The golden standard of diagnose is to find Birbeck particle by electron microscopy, while the examination is seldom carried out in clinic [6]. Besides, the distribution density of Birbeck particle varies widely in different tissues and affected organs. The main immunohistochemical manifestation is S-100 protein and CD1a (+). Langerin (CD207), a new monoclonal antibody of LC-specific C-type lectin, is related to the formation of Birbeck particle [7]. Its specificity is higher than CD1a in Langerhans cells. The examination of Langerin can even be tested by the method of electron microscopy. Vimentin, CD45 and ecto-ATPase that hydrolyze the membrane-associated enzyme activity of extracellular ATP and immune related molecules (such as MHCII molecules) are common positive indicators as well. In addition to the typical histopathologic evidences, the case also had expressed CD1a, CD68 and S-100. Taken together, all these evidences indicated a clear and specific diagnosis.

The clinical manifestation varies widely due to the differences among age of onset, the proliferation rate of Langerhans cells and the involved tissues and organs [8]. The research by Braier et al. [9] indicated that the incidence rate of rashes in LCH was about 24%. The patients showed limited invasive nodules and plaques or generalized seborrheic dermatitis-like rashes, which mainly located at torso, scalp, hairline, retroauricular area, skin folds, externalia and etc. Face and feet may also be involved. Besides, it may manifest as ulcers, scabby and granuloma at crissum, groin, armpit or other frictional area as well. Oral and genital mucosae are the most common involved regions. Some patients can appear xanthoma disseminatum that may occur at oral mucosa and conjunctiva. Rash is a common and specific symptom in LCH, and different period rashes which repeatedly occur in batches can be simultaneous developed at a single patient. It's also the initial symptom which promotes the patient to see a doctor in most cases. The rashes can occur together with other organ damages or appear as the only symptom. Sometimes the rashes can be complicated with malignancy [10].

LCH is easy to be misdiagnosed in clinic. The case in this article has been misdiagnosed as “eczema”, “candidiasis”, “hailey-hailey disease (suspicious)”, “seborrheic dermatitis” and etc. In general, the rash could be identified in the following diseases: seborrheic dermatitis (the lesions of LCH is similar to seborrheic dermatitis sometimes, but the latter doesn't have systemic symptoms and hepatosplenomegaly in infancy), eczema, candidiasis, hailey-hailey disease, pemphigus vegetans, Extramammary Paget's disease and xanthomatosis (LCH should be differentiated from other diseases that may have xanthoma damage when it has the similar damage). The others may have hyperlipaemia or other basic diseases symptoms, while seldom have obvious systemic symptoms or bone injury. Bone marrow and histopathologic examination should be carried out when necessary. It is quite important to identify LCH from Non-Langerhans cell histiocytosis such as juvenile xanthogranuloma, xanthoma disseminatum, acrodermatitis enteropathica, Bloch-Siemens syndrome, mastocytosis, eosinophilic pustular folliculitis, erythema neonatorum toxicum, Wiskott-Aldrich Syndrom, Rosai-Dorfman disease, neonatal pustular melanosis, perinatal listerellosis, neonatal varicella, leukemia, leucoma, myeloma, malignant melanoma, cutaneous Crohn's disease and etc.

The prognosis of LCH is closely related to age of onset, number of involved organs and degree of functional lesion. The prognosis of single-organ involvement is better than multiplied-organ involvement, and the latter has higher case-fatality rate. Adult LCH normally has a good prognosis. The prognosis is poor when the patient has lung, liver, spleen and bone marrow damages and a bad response to the early treatment [11], while it is good when there is only skin and bone infringement. Minority healed children may have sequela of diabetes insipidus, hypophrenia, hypoevolutism, jaw bone dysplasia and etc [12]. A 39-year follow up data in Sweden shows that the overall mortality of LCH patients was about 10% (in children) [13].

Treatments of LCH include surgery, radiotherapy, topical corticosteroids or Mechlorethamine Hydrochloride aqueous solution, thalidomide, systemic chemotherapy and combination therapy. With the progress of chemotherapy, the prognosis of LCH is much better than before. The specific treatment depends on the following factors: the classification, focal or general systematic disease, dysfunction of the mainly affected organs and the age factor.

As LCH isn't infiltrating malignant cells, strong chemotherapy regimen is not recommended in order to avoid severe toxic and side effects. PUVA has also been used for extensive skin lesions, which turns out to have a remarkable effect. Some clinical reports show that the rashes get complete remission with systematic use of isotretinoin [14]. Besides, thalidomide is also effective for the remission of rashes [15].

The patient in this case was performed with systemic chemotherapy and oral isotretinoin, due to the multisystem damage and generalized rash. Besides, the axillary ulcer was excised and given skin-grafting. Then, after a year's follow-up, it turns out that the effect is considerable. But, LCH relapse easily and may accompany with malignant tumor, so it is still necessary to carry out long-time follow-up and observation.
